# LONGITUDINAL ASSOCIATION OF FOOD INSECURITY AND MOBILITY LIMITATIONS AMONG US OLDER ADULTS

**DOI:** 10.1093/geroni/igae098.4233

**Published:** 2024-12-31

**Authors:** Usha Dhakal, Khalil El Asmar, Carlos Mendes de Leon

**Affiliations:** Georgetown University, Washington, District of Columbia, United States; American University of Beirut, Beirut, Beyrouth, Lebanon; Georgetown University, Washington, District of Columbia, United States

## Abstract

Food insecurity among older adults is associated with a range of adverse health outcomes, including cognitive impairment, poor mental health, and mortality. The role of food insecurity in impairments in functional status, such as mobility limitations, remains unclear. Therefore, we used weighted generalized linear models to investigate the association between food insecurity and mobility limitations. Considering the 2014 Health and Retirement Study (HRS) wave as the baseline, we extracted data from an HRS subsample who completed the 2013 Health Care and Nutrition Study (n=6476) and three additional HRS waves through 2020. Mobility limitations scores were based on five items (range:0-5); higher scores indicated more limitations. Food insecurity scores were derived from a validated scale of food access and availability. On average, older adults had difficulty performing at least one mobility task at baseline, and their mobility limitations score increased by 0.5 points per year. After adjustment for demographic and socio-economic factors, greater food insecurity was significantly associated with greater mobility limitations at baseline (β=0.19, 95%CI[.15,.23], p<.0001), but it was not significantly associated with greater increase in mobility limitations over time (β=0.004, 95%CI[.002,.009], p=.22). Associations remained mostly unchanged after controlling for chronic conditions and depressive symptoms. Our findings suggest that food insecurity is associated with mobility limitations, although its role in continued mobility decline over time was small and not statistically significant. Early identification of at-risk older adults for food insecurity may still offer benefits for improved mobility and thereby contribute towards better overall physical functioning and prevent disability onset.

